# Operationalising AI governance in higher education: A stepwise policy-development method

**DOI:** 10.1016/j.mex.2026.104063

**Published:** 2026-07-24

**Authors:** Hai Ninh Nguyen

**Affiliations:** School of International Business and Economics, Foreign Trade University, 91 Chua Lang Street, Lang Ward, Hanoi, Viet Nam

**Keywords:** AI governance, Responsible AI use, Generative artificial intelligence, Policy-development method, AI-use policy, Higher education

## Abstract

The institutional adoption of generative artificial intelligence (AI) requires governance methods that turn use-specific risks into implementable decisions, safeguards, responsibilities and review routines. Existing frameworks provide normative guidance, but few operationalise governance at the level of individual AI-use scenarios. This article presents the Higher Education Artificial Intelligence Policy Development Method (HE-AIPD), a stepwise method that enables cross-functional teams to develop institutional AI-use policies. HE-AIPD converts AI-use evidence into a reusable governance package comprising decision rules, risk safeguards, policy clauses, role responsibilities, implementation tools and a review cycle. The method also includes a traceability design linking use cases to decisions, clauses, roles and tools. Content validity was assessed through an expert walkthrough with 20 participants using a 4-point scale across 12 components and 5 criteria, yielding an overall S-CVI/Ave of 0.882.

Operationalises institutional AI governance by converting AI-use scenarios into decisions, safeguards, clauses, roles, tools and review routines.

Provides reusable governance outputs: scope sheet, use-case inventory, decision matrix, risk–safeguard map, clause bank, implementation toolkit and review cycle.

Includes reproducible content-validity calculations based on expert walkthrough data deposited in Mendeley Data.

## Graphical abstract

**Figure:**
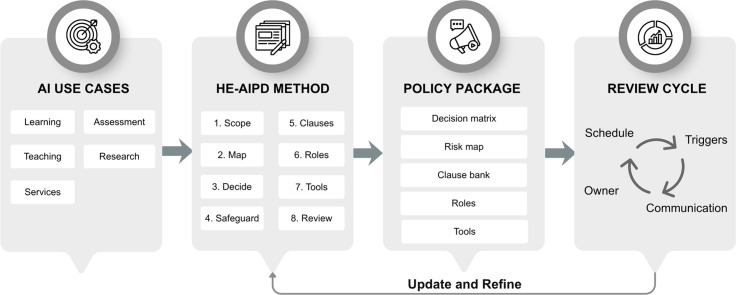

**Specifications table**

**Table:** 

**Subject area**	Computer Science
**More specific subject area**	AI governance; responsible AI use; institutional AI-use policy development; AI risk–safeguard mapping; generative AI in higher education
**Name of your method**	Higher Education Artificial Intelligence Policy Development Method (HE-AIPD)
**Name and reference of original method**	Not applicable. HE-AIPD is presented as a newly developed stepwise method rather than a customization of an existing published method.
**Resource availability**	Supplementary resources include an anonymised expert walkthrough dataset, tidy CVI dataset, CVI results and revision log, worked example, reusable policy package and traceability link tables. All files are deposited in Mendeley Data: 10.17632/9sxm7t8phb.1

## Background

Generative artificial intelligence is increasingly embedded in institutional activities spanning learning support, assessment design, teaching preparation, research assistance, student services and administrative automation [[1], [2], [3]]. Institutions are expected to enable responsible use while managing risks related to academic integrity, data protection, fairness, accountability and tool reliability [[4], [5], [6], [7]]. Despite growing recognition of this responsibility, many institutions lack operationalised governance methods. Institutional guidance documents provide reference points for emerging higher education AI policies [8,9], while broader AI ethics and responsible AI frameworks specify principles such as transparency, accountability, human oversight, privacy, fairness and explainability [[10], [11], [12], [13], [14]]. However, these sources typically do not specify how a cross-functional team should move from use-case evidence to implementable policy decisions, safeguards and review routines [[4], [5], [6], [7], [8], [9], [10], [11], [12], [13], [14]].

HE-AIPD was developed to address this implementation gap. Intended for cross-functional teams that need to develop or revise institutional AI-use policies in higher education, the method does not require a deep technical background; it provides a structured sequence of governance activities and templates that convert AI-use scenarios into policy outputs. In doing so, it contributes to the emerging literature on AI governance operationalisation [[4], [5], [6], [7], [8], [9],[11], [12], [13], [14]] by offering a reproducible, traceable and formatively validated procedure.

## Method details

HE-AIPD is a stepwise policy-development method for operationalising institutional AI governance in higher education. It can be applied when an institution is creating an AI-use policy for the first time, revising an existing policy in response to changed conditions, or extending current policies to cover new AI tools, user groups or risk areas. The method is designed to be reproducible: each step specifies required inputs, a governance action and a practical output. The eight steps proceed iteratively; later steps may prompt a return to earlier ones as the team encounters edge cases, new evidence or stakeholder feedback.

### Intended users and required inputs

The method is designed for cross-functional AI-governance and policy-development teams. Typical users include academic leaders, quality assurance staff, academic integrity officers, teaching and learning developers, information technology and data-protection staff, legal or compliance advisors and student representatives. External consultants may support the process; however, the method requires institutional knowledge of current governance structures, assessment regulations and stakeholder concerns that is best provided by internal team members.

Minimum inputs include: existing academic integrity and assessment regulations; data-protection rules; research ethics guidance; current or anticipated AI use cases; stakeholder concerns; approved, restricted or prohibited tool lists where available; and relevant national or international AI governance frameworks. No specialist technical background is required to apply the method.

### Overview of the AI-governance method

**Fig. 1:**
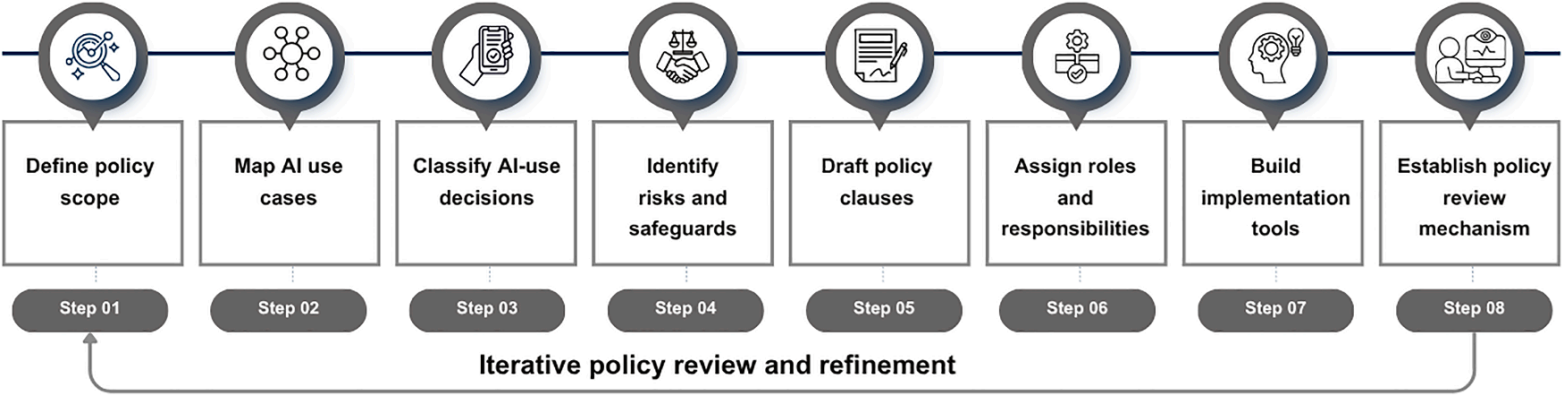
The eight-step workflow of the HE-AIPD method.

The method proceeds through eight iterative steps (Fig. 1), each generating a practical governance output that can be reused or adapted. Beginning with policy-scope definition, the steps then map AI-use scenarios, classify them into governance decision categories, identify risks and safeguards, draft policy clauses, assign roles and responsibilities, build implementation tools and establish a policy review mechanism.

Fig. 1 should be read as the operational backbone of the method. The first three steps establish the governance boundary and classify AI-use scenarios; Steps 4–7 convert this classification into safeguards, policy clauses, role assignments and implementation tools; Step 8 closes the cycle with a review mechanism that maintains policy relevance over time.

The outputs in Table 1 are cumulative. A use-case inventory without decision categories does not yet support policy writing; a decision matrix without safeguards does not yet manage risk; and a clause bank without role assignments is unlikely to be implemented. The method is therefore designed so that each step's output feeds directly into the next.

**Table 1:** HE-AIPD steps, actions and outputs.

Step	HE-AIPD step	Main action	Output
1	Define policy scope	Set the institutional, faculty, programme, course or unit boundary; define user groups and activities covered.	Policy scope sheet
2	Map AI use cases	List current, planned and plausible AI uses across learning, assessment, teaching, research, services and operations.	AI use-case inventory
3	Classify AI-use decisions	Assign each use case to a policy decision category using its purpose, risk level, assessment context and data sensitivity.	AI-use decision matrix
4	Identify risks and safeguards	Map each use case to risks and control measures related to integrity, assessment validity, privacy, equity, bias and accountability.	Risk–safeguard map
5	Draft policy clauses	Convert decisions and safeguards into policy wording for acceptable use, disclosure, assessment, staff use, data protection and review.	Policy clause bank
6	Assign roles and responsibilities	Specify responsibilities for students, lecturers, programme leaders, QA/integrity offices, IT/data protection and academic leadership.	Role responsibility table
7	Build implementation tools	Create templates, checklists, guidance, training plans, tool lists and reporting forms that make the policy usable.	Implementation toolkit
8	Establish policy review mechanism	Define the policy owner, scheduled review, triggered review events, evidence to review and communication route.	Policy review cycle

### Evidence-to-governance translation logic

**Fig. 2:**
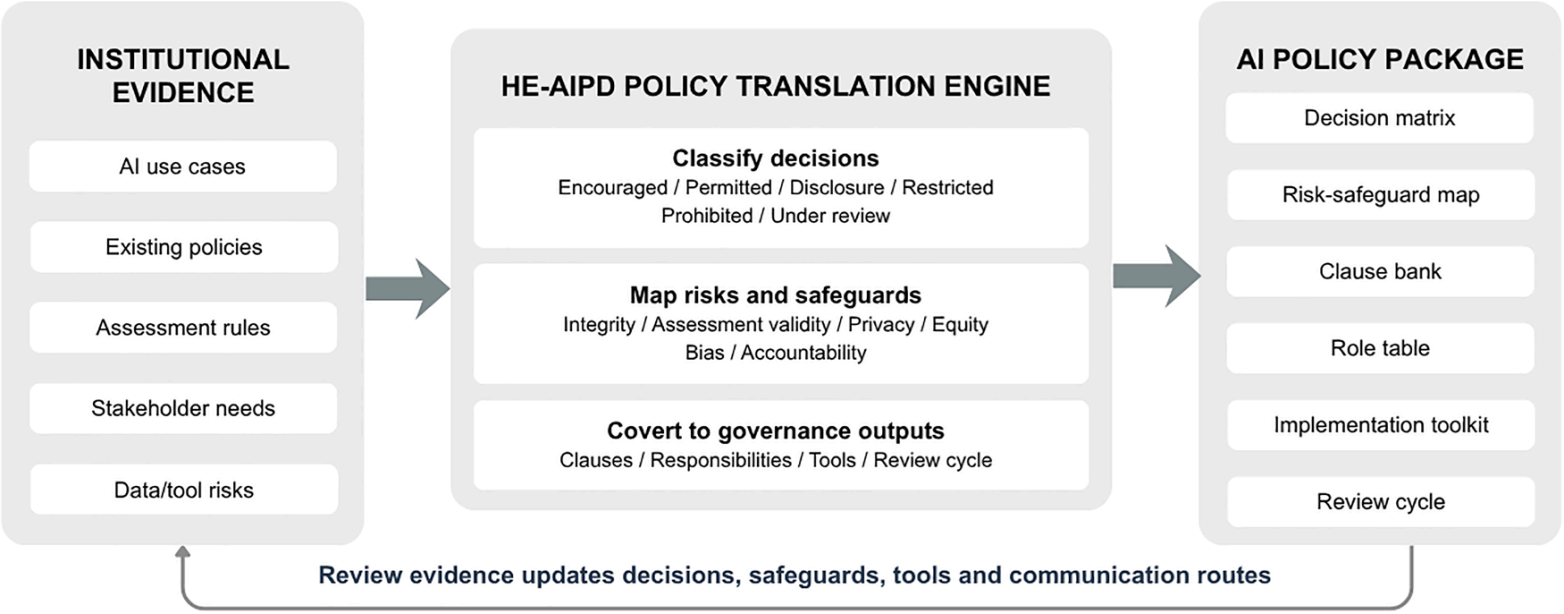
Evidence-to-governance logic of the HE-AIPD method.

The central logic of HE-AIPD is that institutional evidence drives policy outputs rather than remaining as background information. Fig. 2 represents the method as a translation engine: evidence enters on the left, is converted through classification, safeguard mapping and clause drafting, and exits as a governance package on the right.

The left-hand evidence block in Fig. 2 defines what the team must gather before drafting policy text. The central engine shows the three conversion operations that make the method reproducible: classification converts scenarios into decision categories; safeguard mapping converts categories into risk controls; and clause drafting converts controls into enforceable text. The right-hand governance package shows the set of outputs that constitutes a complete institutional AI-use policy.

### Decision categories

Step 3 converts AI-use scenarios into governance decision categories. These categories are intentionally more granular than a simple allow/prohibit distinction. They reflect common institutional policy positions while remaining adaptable to local governance structures.

Table 2 is used at the point where a use case becomes a governance decision. The categories are not intended to rank AI use from good to bad. Instead, they specify the level of permission, disclosure, oversight and safeguarding that the institution commits to for each classified use case.

**Table 2:** AI-use decision categories used in HE-AIPD.

Decision category	Meaning	Illustrative example
Encouraged	Use is actively supported because it contributes to learning, accessibility, feedback, productivity or responsible experimentation.	AI-supported brainstorming, practice questions or language improvement where the task does not assess unaided production.
Permitted	Use is allowed without special disclosure where it does not materially affect assessed output, data protection or decision integrity.	A lecturer uses AI to generate initial teaching ideas and checks all content before use.
Permitted with disclosure	Use is allowed but the user must disclose type, purpose and extent of AI assistance.	A student uses AI for translation or language editing in an assessment where such assistance is allowed.
Restricted	Use is allowed only under specified conditions, safeguards and oversight.	AI-supported formative feedback, student chatbot support or research data coding with human review and data controls.
Prohibited	Use is not allowed because it undermines assessment validity, privacy, fairness, safety or accountability.	Use of AI during a closed-book examination or uploading identifiable student data to a public AI tool.
Under review	Use cannot yet be classified because evidence, legal status, risk or institutional capacity is insufficient.	A new automated grading or learning analytics tool awaiting governance review.

### Detailed AI-governance procedure

A typical implementation can be organised as three to five facilitated working sessions: scoping and use-case mapping; decision classification and risk–safeguard mapping; clause and role drafting; implementation-tool design; and review-cycle approval. Each decision should be documented with a short rationale, responsible role and review trigger so that the resulting policy package remains auditable and revisable.

• Step 1: Define policy scope
  ° The team specifies the level of policy application, the user groups covered, the activities included and the relationship between the AI-use policy and existing rules. A policy may apply at institutional, faculty, programme or course level. Scope definition prevents policy drift, reduces duplication with existing policies and limits scope creep during later drafting.
  ° Practical output: the policy scope sheet should record the institutional level, covered user groups, covered activities, excluded activities, related policies and escalation route. This prevents later disputes about whether a specific activity, tool or user group falls within the policy.
• **Step 2: Map AI use cases**
  ° The team compiles an AI use-case inventory. Use cases should be described in practical terms: user group, activity area, tool type, data type, whether the output affects assessment or decision-making, and current or anticipated frequency of use. The inventory should include observed uses, planned uses and plausible future uses based on technology trends.
  ° Practical output: each use case should be recorded with a short scenario description, user group, activity area, data type, assessment relevance, tool status and human-review requirement. This makes later classification and safeguard mapping more precise.
• **Step 3: Classify AI-use decisions**
  ° Each use case is classified using the decision categories in Table 2. Classification should consider task purpose, assessment stakes, data sensitivity, potential harm, equity implications, accountability requirements and tool maturity. Borderline cases should be flagged for deliberation rather than arbitrarily assigned to a permissive category.
  ° Practical output: the decision matrix should record the decision category and the reason for classification. Borderline cases should remain under review rather than being forced into a permissive or prohibitive category without sufficient evidence.
• **Step 4: Identify risks and safeguards**
  ° For each use case, the team identifies risks related to integrity, assessment validity, privacy, bias, hallucination, equity, accessibility, accountability, data leakage, dependency on proprietary tools and regulatory compliance. Each identified risk is paired with at least one safeguard and one responsible role.
  ° Practical output: each identified risk should have at least one safeguard and one responsible role. A risk without a safeguard is only a warning; a safeguard without an owner is unlikely to be implemented. Risks should be prioritised as high, medium or low according to likely harm, reversibility, data sensitivity and assessment stakes. High-risk items should not be classified as permitted or restricted unless an owner, safeguard and review trigger have been specified.
• **Step 5: Draft policy clauses**
  ° The team converts decisions and safeguards into policy clauses. Clause writing should avoid broad statements that cannot be implemented. Each clause should state who is covered, what is permitted or required, under what conditions, and with what consequences for non-compliance.
  ° Practical output: each clause should be linked back to the decision category and safeguard that justified it. This avoids generic clauses and supports later review when tools, regulations or assessment practices change.
• **Step 6: Assign roles and responsibilities**
  ° Policy implementation requires role allocation. Students are responsible for following task-level AI rules and disclosing assistance where required. Lecturers design assessment rules, communicate course-level policies and report incidents. Programme leaders coordinate consistency across courses. Quality assurance and academic integrity offices maintain institutional oversight. Information technology and data-protection staff manage tool approval and data-handling rules.
  ° Practical output: responsibilities should distinguish between decision ownership, implementation, monitoring, escalation and communication. This reduces the risk that AI-use governance becomes everyone's general responsibility and no one's specific task.
• **Step 7: Build implementation tools**
  ° The policy is translated into implementation tools. Central tools include an approved/restricted tool list, data-use guidance, disclosure policy, training plan and reporting form. Course-level tools include assessment task sheets with AI-use guidance, disclosure templates, course outline inserts and student-facing summaries.
  ° Practical output: the toolkit should include both central governance materials and course-level user-facing materials, which also support institutional AI literacy by helping staff and students interpret acceptable use, disclosure expectations and data-handling requirements [[15], [16], [17]]. This step was the lowest-rated component in the expert walkthrough, indicating that implementation tool design is the most operationally demanding aspect of the method.
• **Step 8: Establish policy review mechanism**
  ° The team defines the policy owner, scheduled review cycle, triggered review events and communication routes. Triggered reviews should occur when new AI tools are adopted, an incident occurs, regulatory changes take effect or stakeholder feedback indicates that the policy is not working as intended.
  ° Practical output: the review mechanism should specify scheduled review, triggered review, evidence sources, decision authority and communication channels. Without this mechanism, an AI-use policy may become outdated within months of publication.

### Worked example and traceability

**Fig. 3:**
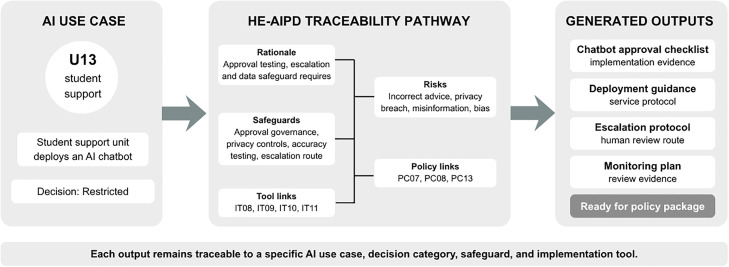
Use-case to policy-package traceability.

The worked example demonstrates how a single AI-use scenario can be traced through the method. The example in Fig. 3 uses an AI chatbot for student support. This use case is classified as restricted use, linked to risks of over-reliance and privacy, safeguarded by human-review requirements and data-handling rules, converted into policy clauses, assigned to IT and student services roles, supported by a disclosure template and subject to triggered review when the tool changes.

Fig. 3 also clarifies why the worked example serves as more than an illustration: it demonstrates the audit trail required for institutional use, showing which risks justified the restricted classification, which safeguards were mapped to those risks, which clauses operationalised the safeguards, and which implementation tools were built to support compliance.

### AI-governance architecture and review cycle

HE-AIPD separates stable institutional governance boundaries from local implementation. The institutional level defines common decision categories, prohibited uses, data rules, tool-approval principles and escalation routes. Faculty, programme or service units adapt these within defined limits for their specific contexts.

**Fig. 4:**
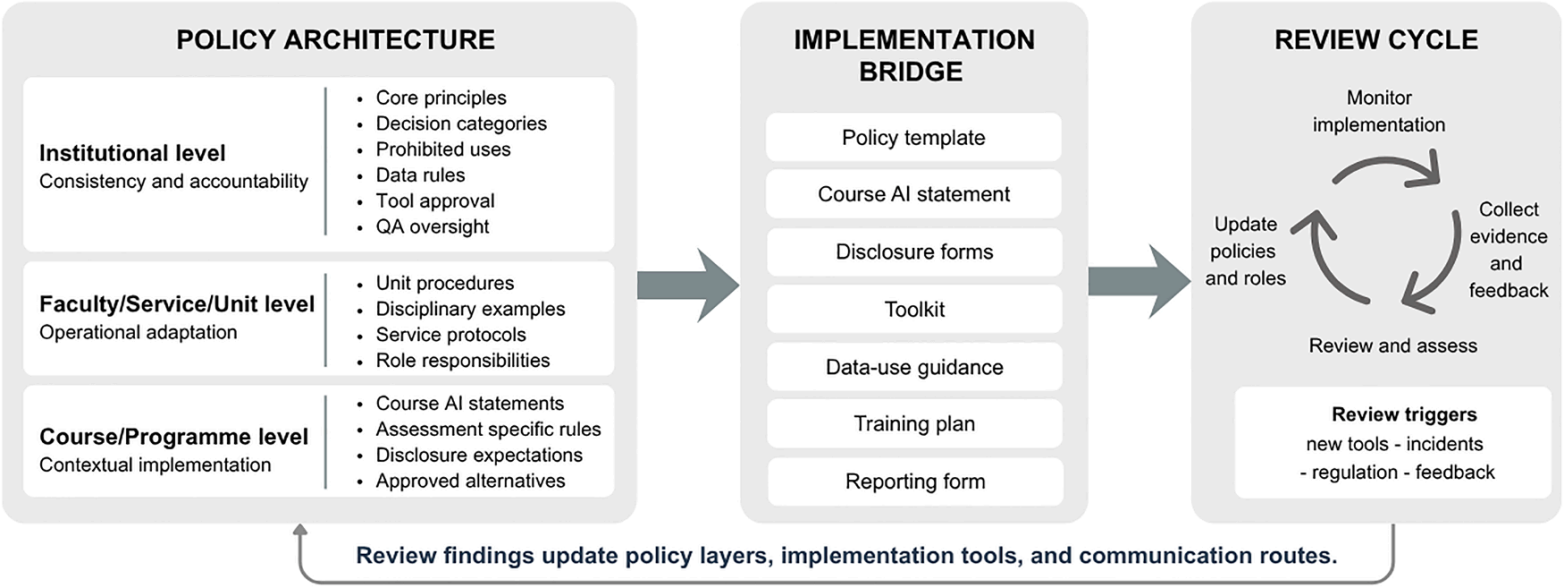
AI-governance architecture and review cycle.

Fig. 4 separates three implementation layers: the institutional layer protects consistency by defining common categories, prohibited uses and data/tool rules; faculty, programme or service units adapt this framework to local conditions; and courses implement both institutional and faculty decisions through task-level guidance and disclosure requirements. The review cycle runs across all three layers, triggered by incidents, tool changes and regulatory updates.

## Method validation

HE-AIPD was formatively evaluated through an expert walkthrough and content-validity assessment. The purpose of this evaluation was not to claim implementation effectiveness, but to test whether the procedure and its components were judged sufficiently relevant, clear, feasible, complete and transferable by a cross-functional expert panel.

**Fig. 5:**
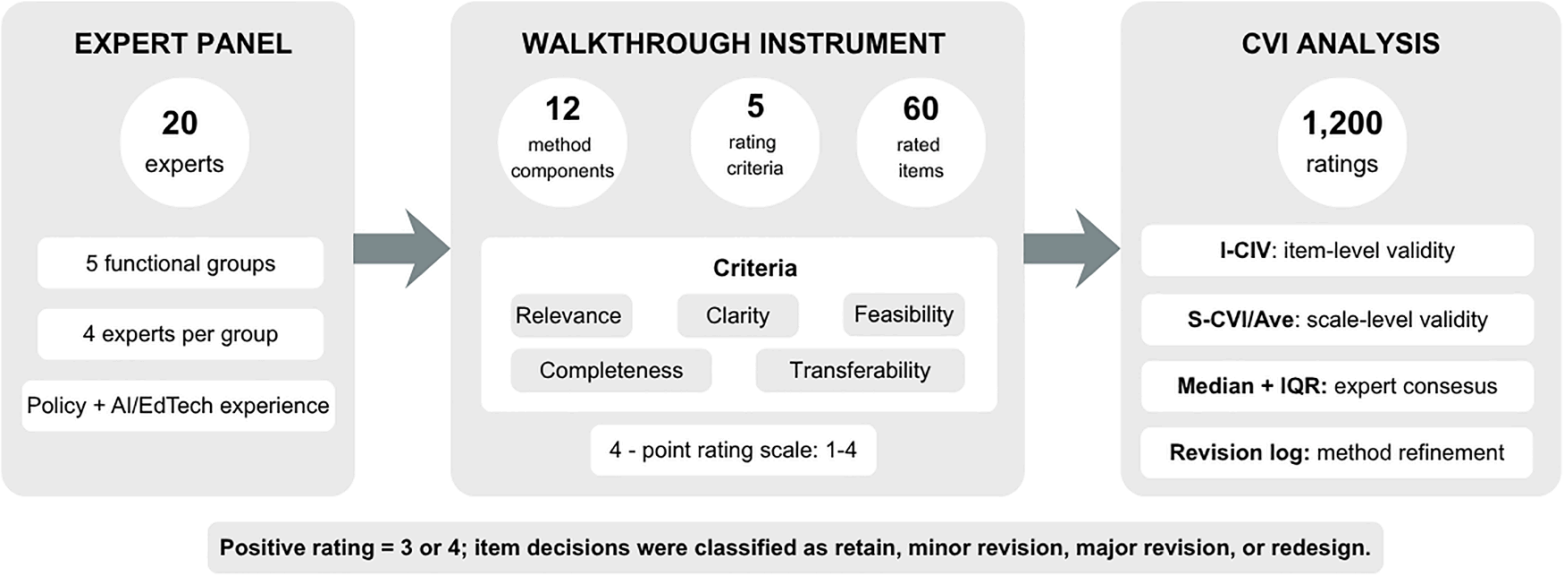
Expert walkthrough and CVI design.

Fig. 5 should be read from left to right: the expert panel provides judgemental evidence; the walkthrough instrument converts the method into 60 component-by-criterion items; and the CVI analysis converts the item ratings into component-level and scale-level validity indices.

Table 3 reports the validation design. The five criteria were selected to evaluate different aspects of method usability: relevance assessed fit to the governance problem; clarity assessed whether the procedure steps and outputs were understandable; feasibility assessed whether the method could realistically be applied in a higher education setting; completeness assessed whether the procedure covered the necessary governance activities; and transferability assessed whether the method could be applied across different institutional contexts.

**Table 3:** Expert walkthrough design.

Element	Description
Experts	20 experts from five functional groups
Functional groups	Academic integrity/examination/ethics; educational technology and AI in education; governance and quality assurance; IT/data protection and cybersecurity; AI tools and learning analytics
Components rated	12 method components
Criteria	Relevance, clarity, feasibility, completeness and transferability
Rating scale	4-point scale, where ratings of 3 or 4 were treated as positive ratings
Total rating cells	1200 (20 experts × 12 components × 5 criteria)
CVI indices	I-CVI for each rated item; S-CVI/Ave across 60 items
Additional analysis	Median, IQR, item-level decision category and open-comment revision log

Experts were included because of professional experience in at least one of the five functional areas represented in the walkthrough panel. Ratings were completed individually using an anonymised walkthrough instrument. Experts assessed the pre-revision version of the method, and the revision log was produced after the rating and comment data had been reviewed.

Table 4 converts the CVI results into revision decisions. The thresholds were used to separate components that could be retained from those needing minor wording changes, substantial operational clarification or fundamental redesign.

**Table 4:** Item-level CVI decision rules.

Decision	Rule
Retain	Positive ratings ≥ 18 and item median ≥ 3.5
Minor revision	Positive ratings ≥ 16 and item median ≥ 3.0, but not meeting the retain rule
Major revision	Positive ratings between 14 and 15, or positive ratings ≥ 14 with item median < 3.0
Redesign	Positive ratings < 14

### Formulae used for CVI calculation

Let N = 20 experts, C = 12 HE-AIPD components, K = 5 rating criteria, and R_eck be the original 1–4 rating given by expert e for component c on criterion k.

• Positive-rating recoding: P_eck = 1 if R_eck ≥ 3; otherwise P_eck = 0.
• Item-level content validity: I-CVI_ck = sum(P_eck) / N.
• Scale-level average content validity: S-CVI/Ave = sum(I-CVI_ck) / (C × K).
• Total positive ratings: sum(P_eck) across all experts, components and criteria.
• Median and IQR were calculated from the original 1–4 ratings for each component × criterion item.

Table 5 summarises the validation dataset and the main scale-level results. The dataset contains the full 20 × 12 × 5 rating structure; therefore, the S-CVI/Ave is calculated from all 60 component-by-criterion items.

**Table 5:** Overall CVI summary.

Metric	Value
Number of experts	20
Number of expert groups	5
Rating components	12
Rating criteria per component	5
Total rating cells	1200
S-CVI/Ave across 60 rated items	0.882
Average item median	3.4
Average item IQR	1.0
Item decisions: Retain	22
Item decisions: Minor revision	35
Item decisions: Major revision	3
Item decisions: Redesign	0
Positive ratings	1058
Overall positive rating rate	88.2%

Table 6 translates the item-level CVI and median values into revision decisions. The distribution indicates that most items were acceptable with either no revision or minor revision, while three items required major revision.

**Table 6:** Item-level decision distribution.

Decision	Number of items	Percentage
Retain	22	36.7%
Minor revision	35	58.3%
Major revision	3	5.0%
Redesign	0	0.0%

Component-level decisions were assigned using the most conservative item-level decision within each component rather than by average I-CVI alone. Therefore, a component with a high average I-CVI could still require minor revision if at least one criterion-level item indicated wording, boundary or implementation refinement.

Table 7 is used to identify which parts of the method required refinement. The highest-rated components were the early scoping and use-case mapping steps, whereas implementation-oriented components received lower ratings.

**Table 7:** Component-level CVI summary and revision implications.

ID	Component	Avg I-CVI	Decision	Revision implication
C1	Overall purpose and scope	0.97	Minor revision	Clarify wording and scope boundaries.
C2	Intended users and implementation context	0.92	Minor revision	Clarify implementation contexts and user roles.
C3	Step 1: Define policy scope	0.99	Minor revision	Minor wording refinement.
C4	Step 2: Map AI use cases	0.93	Minor revision	Minor clarification of use-case categories.
C5	Step 3: Classify AI-use decisions	0.89	Minor revision	Clarify boundaries among decision categories.
C6	Step 4: Identify risks and safeguards	0.90	Minor revision	Strengthen risk–safeguard wording.
C7	Step 5: Draft policy clauses	0.83	Minor revision	Make assessment, disclosure, feedback and data clauses more operational.
C8	Step 6: Assign roles and responsibilities	0.87	Minor revision	Clarify ownership across institutional, faculty/programme and course levels.
C9	Step 7: Build implementation tools	0.77	Major revision	Add central tools, course-level templates and phased implementation guidance.
C10	Step 8: Establish policy review mechanism	0.83	Minor revision	Add review triggers, escalation route, update mechanism and communication route.
C11	AI-use decision categories	0.87	Minor revision	Clarify permitted with disclosure, restricted, prohibited and under review boundaries.
C12	Scenario-based demonstration and reusable outputs	0.81	Minor revision	Strengthen worked example and reusable outputs.

The validation results indicate that HE-AIPD achieved good scale-level content validity, although expert ratings varied across components. The overall S-CVI/Ave was 0.882, and 1058 of 1200 rating cells were positive. The lowest-rated components were Step 7 (Build implementation tools; Avg I-CVI = 0.77) and Scenario-based demonstration and reusable outputs (Avg I-CVI = 0.81). Three items required major revision; no items were rated as requiring redesign.

The revision log therefore focused on strengthening implementation tools, clarifying central-local adaptation, adding triggered-review events, expanding examples for decision categories and making policy clause guidance more operational. These revisions informed the current version of the method presented in this article.

## Limitations

• HE-AIPD is an AI-governance support method, not a legal compliance instrument. Institutions should adapt the method to applicable legal, regulatory and institutional requirements and obtain appropriate legal or data-protection advice before finalising AI-use policies.
• The validation reported in this article is based on a formative expert walkthrough. It provides content-validity evidence for the method components but does not demonstrate field effectiveness, implementation outcomes or long-term governance impact.
• The worked example is illustrative. Actual policy decisions and implementation tools may vary according to local governance structures, assessment regulations, student-support models, procurement processes, data-protection rules and stakeholder input.
• HE-AIPD is not a psychometric scale. CVI was used to assess expert judgement of method relevance, clarity, feasibility, completeness and transferability, not to validate a latent construct or produce an institutional readiness score [[18], [19], [20]]. The validation evidence should accordingly be interpreted as formative evidence of method quality rather than as a predictive measure of implementation success.
• Because AI tools, regulation and institutional practice change quickly, HE-AIPD should be used as an iterative method rather than a one-time policy-production exercise. Institutions should conduct scheduled reviews and triggered reviews when major tools, regulations, incidents or stakeholder practices change.

## Related research article

None.

## Ethics statements

This work involved human participants through a low-risk expert consultation for the development and content-validity assessment of the Higher Education Artificial Intelligence Policy Development Method (HE-AIPD). The activity was reviewed under the applicable institutional procedure and confirmed as exempt from full ethics review. Participation was voluntary and involved adult higher education specialists acting in an individual professional capacity. Informed consent was obtained from all participants. No students, vulnerable participants, clinical intervention, deception, sensitive personal data, or student-level data were involved. Expert ratings and comments were anonymised and reported only in aggregate form; no higher education institution was audited, named, ranked, or evaluated. The principal investigator remained responsible for consent, anonymisation, secure data handling, and compliance with institutional and journal requirements.

## Declaration of generative AI and AI-assisted technologies in the writing process

During the preparation of this work, the author used AI-assisted tools to support drafting and language refinement. After using these tools, the author reviewed and edited the content as needed and takes full responsibility for the content of the publication.

## CRediT authorship contribution statement

**Hai Ninh Nguyen:** Conceptualization, Methodology, Validation, Investigation, Data curation, Formal analysis, Visualization, Writing – original draft, Writing – review & editing, Project administration.

## Declaration of interests

The author declares that he has no known competing financial interests or personal relationships that could have appeared to influence the work reported in this paper.

## Data Availability

Data are openly available in Mendeley Data: 10.17632/9sxm7t8phb.1

## Appendix Supplementary materials

Image, application 1
