# Supplementary material for: Operationalising AI governance in higher education: A stepwise policy-development method
Source: MethodsX. 2026 Jul 24;17:104063. doi: 10.1016/j.mex.2026.104063 (PMC13450495; doi:10.1016/j.mex.2026.104063)
Supplement: Supplementary file 1 [file mmc1.docx]

**Supplementary material and/or additional information [OPTIONAL]**

The supplementary package supports reuse and auditability of the method. The files are deposited in Mendeley Data and should be cited using the dataset reference [21]. The package includes anonymised expert walkthrough data, CVI results and revision log, a worked example linking AI-use scenarios to governance outputs, a reusable policy package and traceability link tables in machine-readable format.

**Table 8.** Supplementary material package.

| **File** | **Contents** | **Role in the method article** |
| --- | --- | --- |
| HE-AIPD README.docx | Brief dataset description, file list, CVI summary, anonymisation statement and reuse notes. | Orientation for reuse |
| HE-AIPD Raw Responses and Tidy CVI.xlsx | Anonymised expert walkthrough responses and analysis-ready tidy CVI data. | Raw and analysis-ready validation data |
| HE-AIPD CVI Results and Revision Log.xlsx | Item-level, component-level and overall CVI results, decision rules and revision log. | Validation evidence and method refinement |
| HE-AIPD Worked Example.xlsx | Worked example linking AI-use scenarios to decisions, risks, safeguards, clauses, roles, tools and review outputs. | Traceability demonstration |
| HE-AIPD Policy Package.xlsx | Reusable policy clauses, role responsibilities, implementation tools and review-cycle elements. | Reusable AI-governance outputs |
| HE-AIPD Traceability Link Tables.xlsx | Machine-readable long-format links connecting use cases, policy clauses, implementation tools and policy-package items. | Auditability and reuse |
